# Involvement of the calcitonin gene-related peptide system in the modulation of inflamed uterus contractile function in pigs

**DOI:** 10.1038/s41598-022-23867-6

**Published:** 2022-11-09

**Authors:** Barbara Jana, Jarosław Całka, Małgorzata Sikora, Katarzyna Palus

**Affiliations:** 1 Division of Reproductive Biology, Institute of Animal Reproduction and Food Research of the Polish Academy of Sciences, Tuwima 10, 10-078 Olsztyn, Poland; 2 Department of Clinical Physiology, Faculty of Veterinary Medicine, University of Warmia and Mazury, Oczapowskiego 13, 10-718 Olsztyn, Poland

**Keywords:** Immunology, Neuroscience, Physiology

## Abstract

This study analyzed severe acute endometritis action on myometrial density and distribution of protein gene product (PGP)9.5- and calcitonin gene-related peptide (CGRP)-like immunoreactive nerve fibers and calcitonin receptor-like receptor (CLR) expression, and on CGRP receptor (CGRPR) participation in uterine contractility in pigs. *E. coli* suspension (*E. coli* group) or saline (SAL group) were injected into the uteri, or only laparotomy was performed (CON group). In the *E. coli* group myometrium, a lack of significant changes in PGP9.5 and CGRP innervation patterns and increased CLR protein level were revealed. In all groups, compared to the pretreatment period, human αCGRP increased amplitude in the myometrium, while reducing it in endometrium/myometrium. In the *E. coli* group endometrium/myometrium, human αCGRP lowered amplitude vs other groups. Human αCGRP reduced frequency in CON and SAL groups and enhanced it in the *E. coli* group endometrium/myometrium. The frequency in *E. coli* group increased vs other groups. CGRPR antagonist, human αCGRP8–37, reversed (CON, SAL groups) and eliminated (*E. coli* group) the rise in human αCGRP-induced myometrial amplitude. In endometrium/myometrium, human αCGRP8–37 abolished (CON group) and reversed (SAL group) a decrease in frequency, and reduced the rise in frequency (*E. coli* group) caused by human αCGRP. Collectively, in the myometrium, endometritis did not change PGP9.5 and CGRP innervation patterns and enhanced CLR protein level. CGRPR also mediated in CGRP action on inflamed uterus contractility.

## Introduction

Calcitonin gene-related peptide (CGRP) is a 37 amino-acid sensory neuropeptide belonging to the CGRP family. This family is also composed by calcitonin (CT), calcitonin receptor-stimulating peptide (CRSP), amylin (AMY) and adrenomedullin (AM). To produce biological effects, CGRP binds to the CGRP receptor (CGRPR), which consists of a calcitonin receptor-like receptor (CLR) and receptor activity modifying protein 1 (RAMP1)^1–3^. CGRP and CGRPR are widely distributed in the peripheral and central nervous systems and peripheral organs. For example, these factors participate in nociceptive processing in the central nervous system, peripheral sensory processing, vascular regulation, and inflammatory processes in visceral organs^4,5^.

Under physiological conditions, the presence of CGRP was revealed in the uterus-innervating neurons of the pig paracervical ganglion (PCG)^6^ and dorsal root ganglia (DRGs)^7^. CGRP-immunoreactive (IR) nerve fibers lie within the human^8^, mice^9^, rat^10,11^ and porcine^12^ endometrium and myometrium, and in the myometrial layer, these fibers supply muscle cells and blood vessels. In healthy uteri of women^13^, mice^14^, and rats^15–17^, CGRP has a relaxing action on smooth muscles of the myometrium. It is also reported that in the myometrium, CGRPR is expressed in women^18,19^, CLR and RAMP1 in rats^20^ and CGRP-receptor component protein (CGRP-RCP), a marker of CGRP-receptor expression, is expressed in mice^21^ and rats^22^. Moreover, CGRPR antagonists reduced the CGRP-induced decrease in the contractility of the human^8^ and rat^16^ myometrium. Regulatory functions of CGRP in implantation, trophoblast proliferation and invasion and fetal organogenesis were also reported^2^.

Among uterine diseases in domestic animals and women in the postpartum period, endometritis and metritis are of significant importance. These pathologies may often lead to disturbances in reproductive processes, and may cause reduced animal production profitability^23,24^. Endometritis and metritis are evoked mainly by bacteria, and favoring factors, for example, hard labor and fetal membrane retention, contribute to the occurrence of these diseases^25^. The origin, development and maintenance of uterine inflammation are due to the dysfunction of immunological processes and/or contractility^26,27^. Uterine contractility is reduced or abolished in severe cases of uterine inflammation, which causes the accumulation of inflammatory exudate in the uterine cavity, and disorders of reproductive processes^28,29^. It was reported that prostaglandins (PG)F_2_α^30^, PGE_2_^31^ and PGI_2_^32^ and leukotrienes (LT)C_4_ and LTD_4_^33^ significantly affect the contractile function of an inflamed pig uterus. In cows after parturition, oxytocin increases the contractility of the inflamed uterus, while PGF_2α_ induces an initial drop of this activity followed by an increase^34^.

Regarding the innervation of the inflamed uterus, it is known that in the gilts with endometritis, the numbers of uterine perikarya expressing noradrenaline (NA), neuropeptide Y (NPY) and vasoactive intestinal peptide (VIP) in the CaMG^35^ and PCG^36^ as well as the sets of uterine perikarya containing substance P (SP) and galanin (GAL) in the DRGs^7^ were increased. The participation of NA^31,32,37,38^, acetylcholine (ACh)^39,40^, NPY^41^, somatostatin (SOM)^42^, VIP^43^ and GAL^44^ in the contractility of inflamed pig uterus in connection with the changes in expression receptors for these transmitters were also reported. It was hypothesized that endometritis affects the myometrial innervation pattern by CGRP nerve fibers and the expression of CLR and CGRP action on the contractile function of an inflamed uterus. Recognition of the CGRP role in the contractility of the inflamed uterus will contribute to broadening knowledge of the neurogenic regulation of the inflamed uterus activity and achieving better results in the prevention and treatment of uterine inflammation in animals. In the current study, it was decided to investigate the effect of endometritis in gilts on (1) the density and distribution of nerve fibers stained for the pan-neuronal marker protein gene product (PGP)9.5 and CGRP in the myometrium, (2) the expression levels of CRL mRNA and protein in the myometrium, and (3) the participation of CGRPR in CGRP-elicited uterine amplitude and frequency of contractions. The obtained data from a study performed on the domestic pig model (with a high similarity of anatomical structures and physiological processes to humans) can be referred to human medicine to better the understanding of the uterine inflammation mechanisms^45^.

## Results

### Density and distribution of PGP9.5- and CGRP-like IR nerve fibers

**Figure 1:**
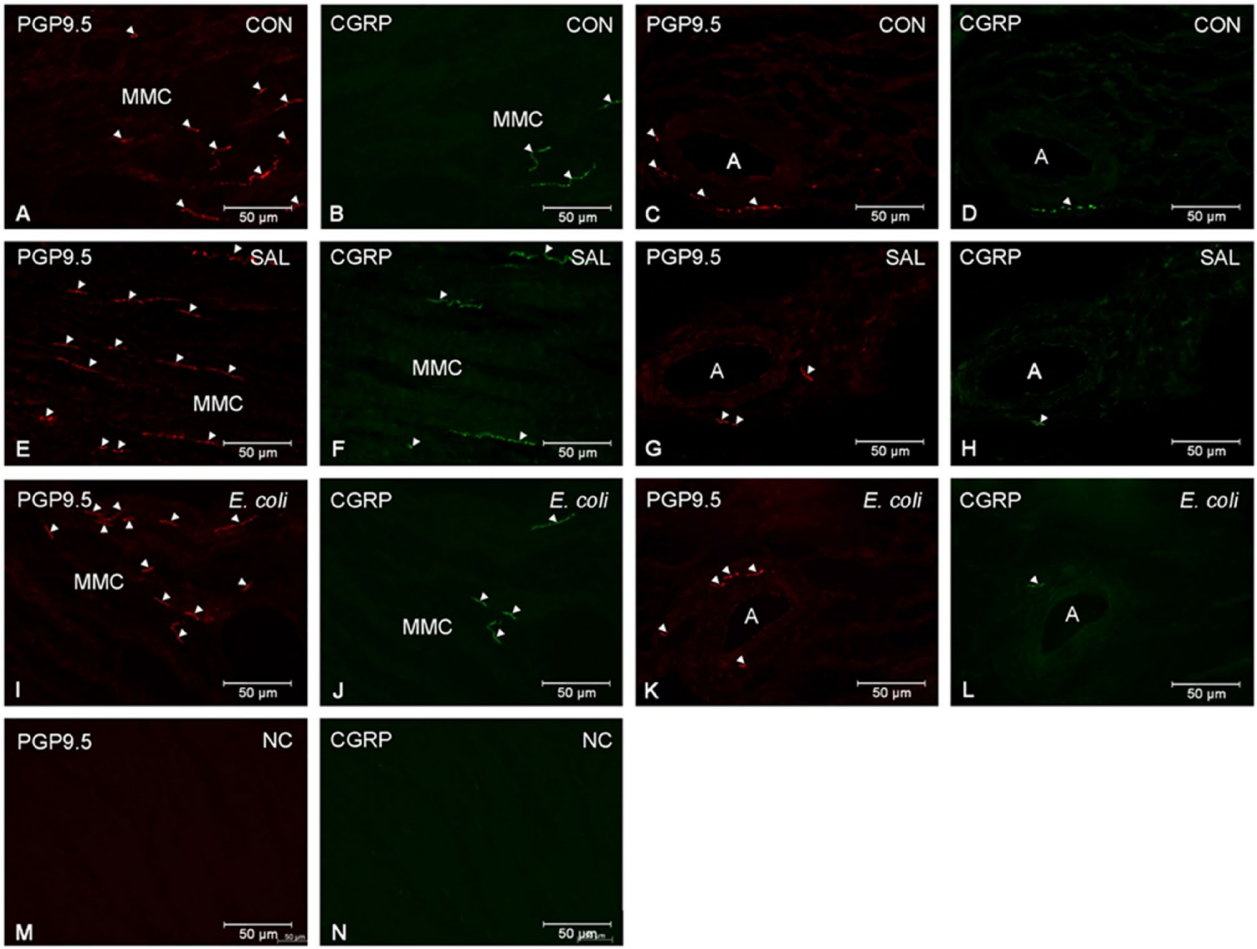
Representative pictures show protein gene product (PGP)9.5- and calcitonin gene-related peptide (CGRP)-like immunoreactive (IR) nerve fibers in the myometrial layer of gilts from the control (CON), saline (SAL) and *E. coli* (*E. coli*) groups. Note that numbers of PGP9.5- and CGRP-like IR nerve fibers around myometrial muscle cells and arteries were similar in the CON (**A–D**), SAL (**E–H**) and *E. coli* (**I–L**) groups. Negative control (NC) for PGP9.5 (**M**) and CGRP (**N**) was obtained by omitting the primary antibodies. *MMC* myometrial muscle cells, *A* artery, *Arrowhead* nerve fiber.

The total numbers of PGP9.5- and CGRP-like IR nerve fibers did not differ significantly in the myometrium of CON, SAL and *E. coli* groups (PGP.9.5: 46.4 ± 2.6, 45.4 ± 2.8, 47.8 ± 4.9; CGRP: 5.5 ± 0.6, 6.1 ± 0.3, 6.9 ± 0.2, respectively). In the CON, SAL and *E. coli* groups, the numbers of fibers expressing PGP9.5 did not differ significantly around the myometrial muscle cells (43.2 ± 3.1, 43.3 ± 3.1, 45.9 ± 4.8, respectively; Fig. 1A,E,I) or blood vessels (4.8 ± 0.5, 5.1 ± 0.7, 6.3 ± 0.3, respectively; Fig. 1C,G,K). A similar situation in the CON, SAL and *E. coli* groups concerned CGRP-like IR fibers near the myometrial muscle cells (3.1 ± 0.6, 2.1 ± 1.1, 1.9 ± 1.2, respectively; Fig. 1B,F,J) and blood vessels (0.7 ± 0.12, 0.5 ± 0.5, 0.6 ± 0.3, respectively; Fig. 1D,H,L).

Moreover, no significant differences were found between the CON, SAL and *E. coli* groups in terms of CGRP-like IR fibers normalized against the total population of PGP9.5-like IR fibers (12.8 ± 1.2%, 13.4 ± 0.8%, 14.8 ± 0.9, respectively).

Immunofluorescent staining of the porcine duodenum, as the positive control, showed PGP9.5- and CGRP-like immunoreactivity in the nerve fibers (Supplementary Fig. 1). PGP9.5- and CGRP-like IR fibers were not present after omitting the primary antibodies (Fig. 1M,N, respectively).

### Expression of CLR messenger RNA

**Figure 2:**
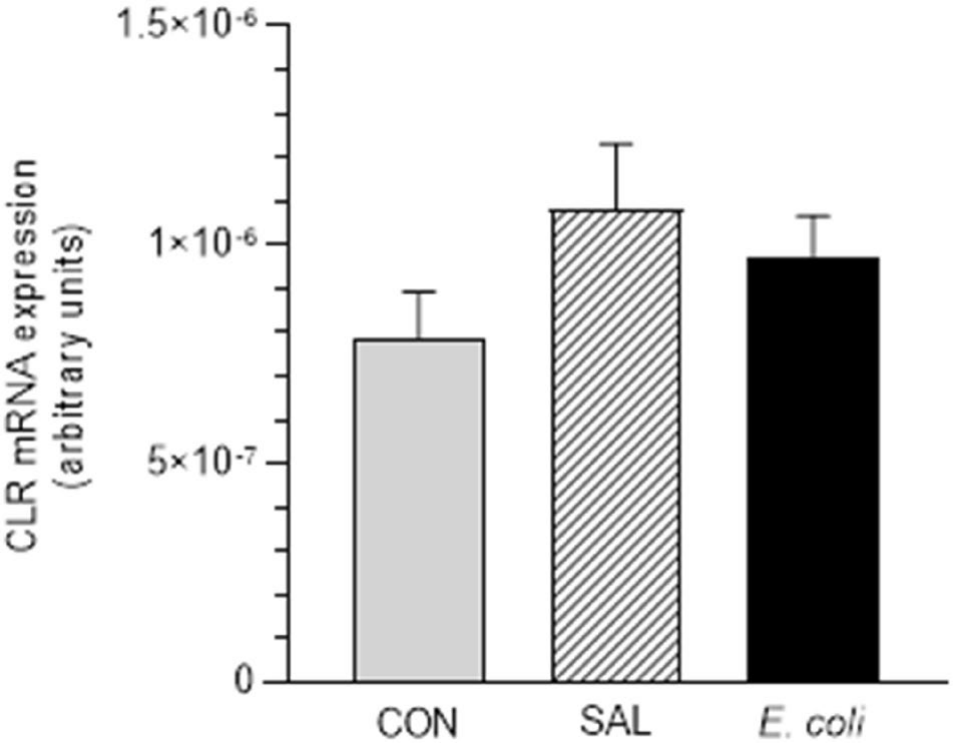
The messenger RNA expression of calcitonin receptor-like receptor (CLR) in the myometrial layer of gilts from the control (CON), saline (SAL) and *E. coli* (*E. coli*) groups, estimated by real-time RT-PCR. Data are expressed as the mean ± SEM (n = 5/gilts in each group). mRNA levels are normalized to glyceraldehyde-3-phosphate dehydrogenase (GAPDH).

No significant differences in the myometrial CLR mRNA expression were revealed between the CON, SAL and *E. coli* groups (Fig. 2).

### Expression of CLR protein

Mice and porcine duodenum utilized as positive controls showed bands of approximately 53 kDa, and they were accepted as CLR protein (Supplementary Fig. 2). The band was not found after not using the primary antibody (data not shown). Western blotting of the porcine myometrium indicated protein bands of approximately 53 kDa for CLR (Supplementary Fig. 3).

**Figure 3:**
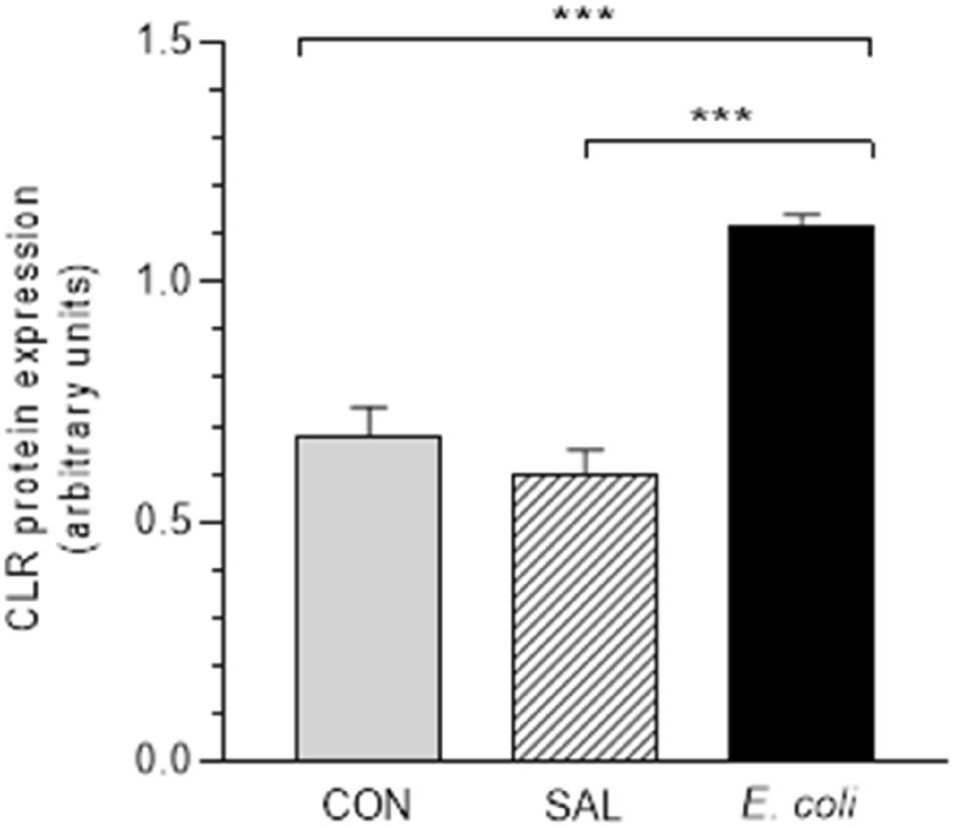
The protein expression of calcitonin receptor-like receptor (CLR) in the myometrial layer of gilts from the control (CON), saline (SAL) and *E. coli* (*E. coli*) groups, estimated by Western blot analysis. Data are expressed as the mean ± SEM (n = 5/gilts in each group). Protein levels are normalized to glyceraldehyde-3-phosphate dehydrogenase (GAPDH). Blot with representative bands for each group is presented in Supplementary Fig. 3. ***P < 0.001 compared between groups.

The CLR protein expression in the myometrium of *E. coli* group was significantly increased in relation to the CON and SAL groups (Fig. 3).

### Distribution of CLR

**Figure 4:**
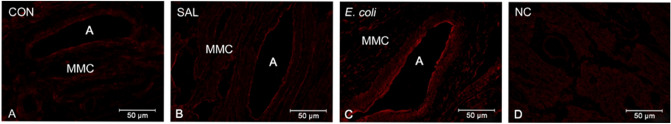
Representative pictures show calcitonin receptor-like receptor (CLR)-like immunoreactivity in the myometrial layer of gilts from the control (CON), saline (SAL) and *E. coli* (*E. coli*) groups. CLR-like immunoreactivity is visible in muscle cells and arteries (endothelium, muscle layer) of the myometrium in the CON (**A**), SAL (**B**) and *E. coli* (**C**) groups. Negative control (NC) for CLR (**D**) was obtained by omitting the primary antibody. *MMC* myometrial muscle cells, *A* artery.

Immunofluorescent staining of the porcine duodenum, as the positive control, showed CLR-like immunoreactivity (Supplementary Fig. 4). CLR-immunoractivity was not visible following omitting of the primary antibody (Fig. 4D). CLR-like immunoreactivity was present in the muscle cells and blood vessels (endothelium, muscle layer) of myometrium in the CON (Fig. 4A), SAL (Fig. 4B) and *E. coli* (Fig. 4C) groups.

### Human α-CGRP (hαCGRP) action on the contractility of uterine strips

#### Comparison of the hαCGRP action in myometrium in the particular groups in relation to the period before its use

**Figure 5:**
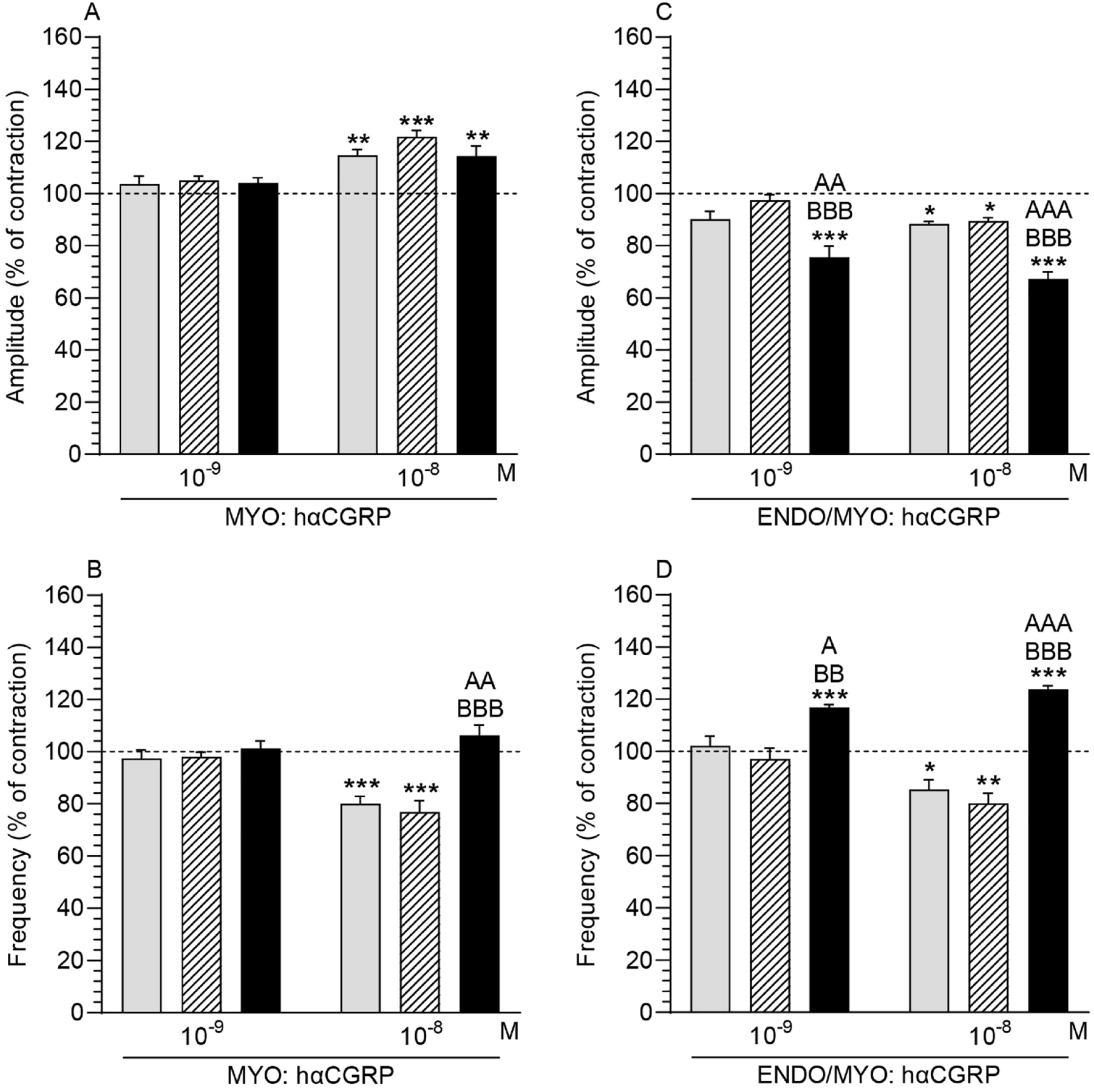
Influence of human α-calcitonin gene-related peptide (hαCGRP) on the contractile amplitude (**A,C**) and frequency (**B,D**) in the myometrium (MYO; (**A,B**)) and endometrium/myometrium (ENDO/MYO; (**C,D**)) strips of gilts from the control (CON; grey bars), saline (SAL; hatched bars) and *E. coli* (*E. coli*; black bars) groups. Data are expressed as the mean ± SEM (n = 5/gilts in each group). The actions of individual hαCGRP doses are depicted as percentages of the baseline (pre-treatment period) contractile amplitude and frequency, taken as 100% (horizontal lines). *P < 0.05, **P < 0.01, ***P < 0.001 compared to the basal value in each group; ^A^P < 0.05, ^AA^P < 0.01 ^AAA^P < 0.001 compared between the CON and *E. coli* groups for the same treatment; ^BB^P < 0.01, ^BBB^P < 0.001 compared between the SAL and *E. coli* groups for the same treatment.

The amplitude in myometrium in response to hαCGRP (10^–8^ M) was significantly increased in the CON, SAL and *E. coli* groups (Fig. 5A). HαCGRP at this dose significantly decreased the frequency in the CON and SAL groups (Fig. 5B).

#### Comparison of the hαCGRP action in myometrium between groups

The frequency in myometrium in the *E. coli* group was significantly enhanced by hαCGRP (10^–8^ M) vs other groups (Fig. 5B). In all groups, the myometrial amplitude did not differ significantly after using hαCGRP (10^–9^, 10^–8^ M) (Fig. 5A).

#### Comparison of the hαCGRP action in endometrium/myometrium in the particular groups in relation to the period before its use

The amplitude in endometrium/myometrium of the CON and SAL groups was significantly reduced by hαCGRP (10^–8^ M), while in the *E. coli* group this effect was exerted by hαCGRP at both doses (10^–9^, 10^–8^ M) (Fig. 5C). HαCGRP (10^–8^ M) significantly decreased the frequency in the tissues of the CON and SAL groups (Fig. 5D). In the *E. coli* group, hαCGRP (10^–9^, 10^–8^ M) significantly enhanced values of this parameter.

#### Comparison of the hαCGRP action in endometrium/myometrium between groups

After using hαCGRP (10^–9^, 10^–8^ M), the amplitude in endometrium/myometrium of the *E. coli* group significantly lowered vs the CON and SAL groups (Fig. 5C). In turn, the frequency in the *E. coli* group in response to hαCGRP (10^–9^, 10^–8^ M) was significantly higher than in other groups (Fig. 5D).

### Human α-CGRP8–37 (hαCGRP8–37, CGRPR antagonist) and hαCGRP action on the contractility of uterine strips

#### Comparison of the hαCGRP8–37 and hαCGRP action in myometrium in the particular groups in relation to the period before their use

**Figure 6:**
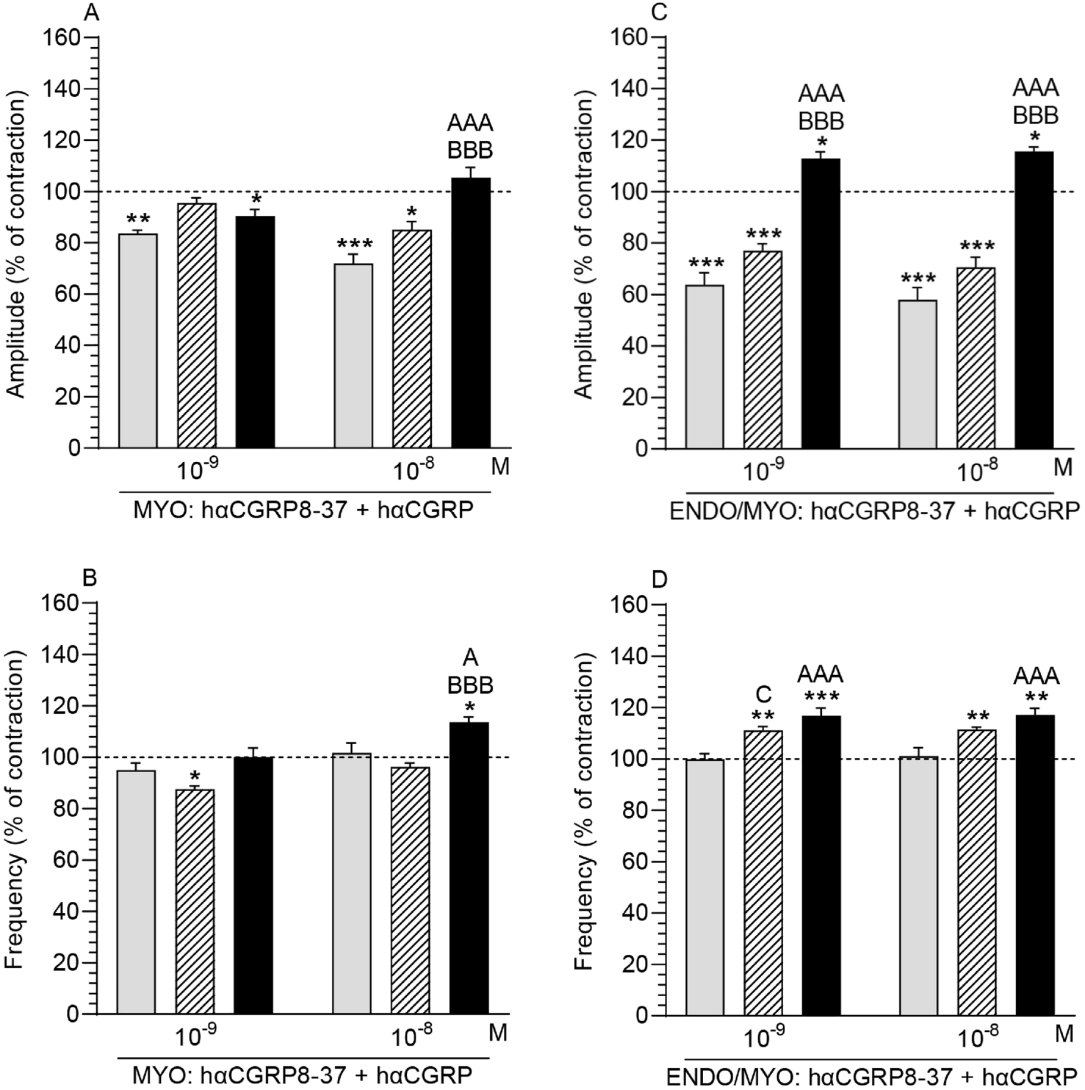
Influence of human α-calcitonin gene-related peptide (hαCGRP) on the contractile amplitude (**A,C**) and frequency (**B,D**) in the myometrium (MYO; (**A,B**)) and endometrium/myometrium (ENDO/MYO; (**C,D**)) stripes of gilts from the control (CON; grey bars), saline (SAL; hatched bars) and *E. coli* (*E. coli*; black bars) groups after the application of human α-calcitonin gene-related peptide receptor (hαCGRP8–37, CGRPR antagonist) (a dose of 10^–7^ M). Data are expressed as the mean ± SEM (n = 5/gilts in each group). The actions of hαCGRP8–37 and individual hαCGRP doses are depicted as percentages of the baseline (pre-treatment period) contractile amplitude and frequency, taken as 100% (horizontal lines). *P < 0.05, **P < 0.01, ***P < 0.001 compared to the basal value in each group; ^A^P < 0.05, ^AAA^P < 0.001 compared between the CON and *E. coli* groups for the same treatment; ^BBB^P < 0.001 compared between the SAL and *E. coli* groups for the same treatment; ^C^P < 0.05 compared between the CON and SAL groups for the same treatment.

After the application of hαCGRP8–37 (10^–7^ M) with hαCGRP (10^–9^, 10^–8^ M), the amplitude in myometrium of the CON group was significantly dropped (Fig. 6A). Similar results were evoked by hαCGRP8–37 and hαCGRP in the myometrium of the SAL group (hαCGRP: 10^–8^ M) and *E. coli* (hαCGRP: 10^–9^ M) groups. HαCGRP8–37 and hαCGRP (10^–9^ M) significantly decreased the frequency in myometrium of the SAL, while the hαCGRP8–37 and hαCGRP (10^–8^ M) significantly increased it in the *E. coli* group (Fig. 6B).

#### Comparison of the hαCGRP8–37 and hαCGRP action in myometrium between groups

The myometrial amplitude (Fig. 6A) and frequency (Fig. 6B) in the *E. coli* group after the application of hαCGRP8–37 and hαCGRP (10^–8^ M) were significantly enhanced vs other groups.

#### Comparison of the hαCGRP8–37 and hαCGRP action in endometrium/myometrium in the particular groups in relation to the period before their use

HαCGRP8–37 (10^–7^ M) and hαCGRP (10^–9^, 10^–8^ M) significantly reduced the amplitude in endometrium/myometrium of the CON and SAL groups while it significantly increased this parameter in the *E. coli* group (Fig. 6C). In the SAL and *E. coli* groups, hαCGRP8–37 together with hαCGRP (10^–9^, 10^–8^ M) significantly increased the frequency in endometrium/myometrium (Fig. 6D).

#### Comparison of the hαCGRP8–37 and hαCGRP action in endometrium/myometrium between groups

HαCGRP8–37 (10^–7^ M) with hαCGRP (10^–9^, 10^–8^ M) caused a significant rise in the amplitude in the endometrium/myometrium of the *E. coli* vs other groups (Fig. 6C). After using hαCGRP8–37 and hαCGRP (10^–9^, 10^–8^ M), the frequency in the endometrium/myometrium of the *E. coli* group was significantly higher than in the CON group (Fig. 6D). HαCGRP8–37 and hαCGRP (10^–9^ M) significantly increased the frequency in the SAL group vs CON group.

## Discussion

Here we present the endometritis influence on the myometrial density and distribution of PGP9.5- and CGRP-like IR nerve fibers and CLR expression as well as the role of CGRP and CGRPR in the contractile function of the porcine inflamed uterus. Results of macroscopic and histopathologic examination of uteri used in the current study were reported earlier^37^. Macroscopically, no inflammatory changes were observed in the endometrium of the CON and SAL groups. In turn, the *E. coli* injections led to the accumulation of inflammatory exudate in the horns, reddening and swelling of the endometrium. Histopathological examination of uterine sections stained with the hematoxylin–eosin method, according to the criteria described previously^46^ did not reveal any changes indicating an inflammatory process in the CON and SAL groups. In the *E. coli* group, a severe acute endometritis has been diagnosed based on the presence of the following changes: edema, hyperemia, damage to the luminal and glandular epithelium and statistically higher number of neutrophils than in the healthy uterus.

The current study found that endometritis did not significantly change the myometrial total population of nerve fibers, as revealed by PGP9.5-like immunoreactivity as well as the numbers of CGRP-like IR fibers (both total number and number in relation to the total population of PGP9.5-like IR fibers). The lack of changes in the myometrial population of CGRP-like IR nerve fibers in response to endometritis is convergent with the unchanged number of uterine perikarya expressing CRGP in the DRGs of pigs suffering from this pathology^7^. Reports also show that inflammation increased the population of PGP9.5- and CGRP-IR nerve fibers in murine vagina^47^ and CGRP-like IR fibers in pig descending colon^48^. It is known that peptides of the CGRP family share a similar secondary molecular structure, and the majority of their functions overlap^2^. Thus, it is possible that the antibody used in the present study to stain CGRP fibers also binds AM, AMY and CRSP. This supposition is based on reports showing the immunoreactivity for AM in perivascular nerve fibers in rat mesenteric artery^49^, AMY in perikarya in cat trigeminal ganglion^50^ and CRSP in pig central nervous system^51^.

As mentioned earlier, CGRPR consists of CLR and RAMP1. Moreover, co-expression of CLR with RAMP2 or RAMP3 constitutes two AM receptors (AM1R and AM2R, respectively)^2,3^. Under physiological conditions, CGRPR was identified in the myometrial layer in women^18,19^. In the myometrium of rats CLR and RAMP1^20^ and CGRP-RCP^22^, as well as of mice CGRP-RCP^21^, were determined. The current report, for the first time, demonstrates the CLR expression pattern in the porcine myometrium of healthy uteri. Moreover, new indications about CLR content in the inflamed uterus are also presented. The antibody used in the current study detected in the uteri the bands at approximately 53 kDa. The data showed CLR bands of 53 kDa in human fetoplacental vessels^52^, of 59 kDa in neuroblastoma derived cell line (SK-N-MC)^53^ and 80 kDa in SK-N-MC cells and cell line derived from human colonic epithelium (Col 29)^54^. The discrepancies in molecular weights can be related to the kind of tissues, differences in protein sample preparation and the kind of antibodies. The CLR mRNA levels were similar in all studied groups. In turn, the intrauterine *E. coli* infusions resulted in a rise in CLR protein expression compared to the CON and SAL groups. The reasons behind the unhanged mRNA level and the increased protein level of CLR in the myometrium of the *E. coli* group are not clear since, in the current experiment, mRNA and protein stability and turnover were not determined. Nevertheless, it is supposed that an elevated CLR protein level may inhibit the gene expression encoded this protein. Similarly to the current results, a rise in CGRPR expression was also found in the mice liver during lipopolysaccharide-induced injury^55^. The CLR protein levels in the CON and SAL groups did not differ significantly, which indicated that the saline injections had no appreciable effect on this receptor expression. While there are literature data showing the effect of sex steroids on the expression level of CGRPR^19^ and components of this receptor (CLR, RAMP1)^20,56^ in the uterus under physiological conditions, there is no information on the factors regulating their level in the uterus with inflammation. The role of PGE_2_ and its EP receptors in the up-regulation of CGRP and RAMP1 expression in the synovium of knee osteoarthritis patients has been^57^. Immunofluorescent staining of the myometrial layer in the CON, SAL and *E. coli* groups, allows for the identification of CLR in the muscle cells and blood vessels cells. Earlier, CLR and RAMP1 were revealed in myometrial myocytes and blood vessels of women^58^ and in the placenta cells of rats^59^. A rise in myometrial CLR protein expression level in the *E. coli* group (present study) may suggest an enhancement in the protein expression of CGRPR, AM1R and AM2R by an inflammatory process. In turn, the authors’ immunofluorescent findings suggest that the muscle and blood vessel cells in the pig myometrium are the sites of CGRP and AM binding in healthy and in the setting of inflammation uteri. However, these assumptions have to be confirmed by simultaneously determining the expression of CLR, RAMP1, RAMP2 and RAMP3 proteins. The correlation designation of both components of CGRPR, AM1R and AM2R can approximate their functional importance for CGRP and AM biological actions. It is known that RAMP1 facilitates the cell-surface expression of CLR and is also essential for the binding of CGRP to the receptor, while RAMP2 and RAMP3 play these functions in relation to AM^2^.

The CGRP influence as a potent dilator is seen in cerebral, coronary and kidney vascular beds. This function is essentially inhibited by the CGRPR antagonist^4,60^. CGRP plays an important role in the course of inflammatory reaction because its release results in edema formation, increased blood flow, and recruitment of inflammatory cells to the local area^3^. Moreover, the relaxing actions of CGRP in the human^61^ and rat^62^ uterine arteries are mediated through CGRPR. In light of the above data, the role of CGRPR in the CGRP action on the blood vessels of the pig myometrium under physiological and inflammatory conditions is possible.

The current study was also devoted to defining the participation of CGRP and its receptor in the contractile function of the inflammatory-changed uterus. As mentioned before, the disturbances in uterine contractility are a significant cause of the origin, development and maintenance of an inflammatory state^26,27^. The use of ACh in the study confirmed the viability and utility of uterine tissues for research. ACh enhanced the amplitude and frequency in healthy uteri (CON and SAL groups). In organs with inflammation (*E. coli* group) in response to ACh, the frequency was increased, while the amplitude was decreased^39^, which is in the line with earlier reports^31,32^.

The current report, for the first time, shows the contractility of healthy pig uteri under the influence of CGRP and the role of CGRPR. Moreover, the current results concern the role of the CGRP system in the contractile function of inflamed uteri. Moreover, the absolutely new results concern meaning of CGRP system in the contractile function of inflamed uteri. In the CON and SAL groups, compared to the time before hαCGRP addition, this neuropeptide increased the amplitude in the myometrium, while it reduced in endometrium/myometrium and the frequency in both kinds of strips. Moreover, direction alternations of these parameters after using hαCGRP8–37 and hαCGRP in the CON and SAL groups showed that CGRPR plays a role in CGRP action on the contractility of the porcine healthy uterus. It is worth adding that in the current research, the values of the contractility parameters and the myometrial expression of CLR mRNA and protein did not differ significantly between the CON and SAL groups. As it was mentioned previously, CGRP, under physiological conditions, decreased the myometrial contractility in humans^13^ and rodents^14–17^. In turn, the effect of CGRP in the gastrointestinal tract was excitatory or inhibitory^63^. Literature data also show the functional role of CGRPR for CGRP in the motility control of the human^58^ and rat^16^ myometrium and the rat colon^64^.

In the myometrium of the *E. coli* group, in relation to the period before hαCGRP application, hαCGRP enhanced the amplitude and did not significantly change the frequency. In turn, in the endometrium/myometrium, a decrease in amplitude and a rise in frequency after using this neuropeptide were found. The direction and level of significance of changes after using hαCGRP8–37 and hαCGRP indicated that CGRPR mediates CGRP action on the contractility of an inflamed uterus, similarly to the healthy uterus. It is important to add that changes in the contractility of inflamed uteri (also healthy) in response to hαCGRP may have also resulted from the action of this peptide via AMY1R (formed by CTR and RAMP1). Moreover, the effect of AM and AMY by CGRPR on the contractile activity of porcine uteri is possible^65,66^. Based on the higher affinity of CGRP8–37 for CGRPR than AMY1R^3^, it is supposed that the changes in uterine contractility noted in the current study after the use of hαCGRP8–37 result mainly from the blockage of CGRPR. Moreover, one cannot exclude that changes of the uterine contractility observed through all experimental groups might be affected by sensibilization and/or desensibilization of the CGRPR. In response to hαCGRP, the amplitude in the endometrium/myometrium of the *E. coli* was lowered and the frequency in both kinds of strips was enhanced vs the CON and SAL groups. These changes in contractility of the inflamed uterus coincided with an augmentation in myometrial CLR protein expression. The lowered amplitude in the endometrium/myometrium and the increased frequency in strips of the *E. coli* group in response to hαCGRP in relation to a healthy uterus could also be due to the indirect influence of this neuropeptide. Existing data demonstrate that CGRP modify the ACh-^14,67^, SP-^15^ and GAL^10^ stimulated uterine contractions in rodents. It should be stressed that ACh^39^ and GAL^44^ markedly change the contractility of porcine inflamed uteri. On the other hand, it is known that the action of CGRP on pig uterine contractility may be dependent on nitric oxide (NO) and PGE_2_, which was indicated earlier^15,68^. The authors’ previous studies reported a significant rise in NO^69^ and PGE_2_^70^ production in the pig uterus with inflammation. These tasks require further research.

Further studies are also necessary to explain the varied CGRP influence on the amplitude in both kinds of strips of the CON, SAL and *E. coli* groups and on the frequency in the latter group. We can only assume that this situation is due to the different innervation of the endometrium and myometrium, and relationships between CGRP and other neurotransmitters in relation to uterine contractility (e.g. SP, GAL and ACh), as mentioned above^10,14,15,67^. Moreover, varied contractility after using CGRP may be dependent on the different content in the endometrium and myometrium of substances modulating this neuropeptide effect. For example, a difference in NO^69^ and PGE_2_^70^ amounts between the particular layers in both healthy and inflamed pig uteri was demonstrated.

Earlier it was reported that NA, ACh, NPY, VIP, GAL and PGE_2_ have lowering action on the amplitude in pig inflamed uterus^31,32,38,39,41,43,44^. Thus, it is possible, that CGRP by decreasing the value of this parameter (present study) is another substance which contributes to the accumulation of inflammatory exudate inside the uterine lumen. In parallel we have now demonstrated that CGRP increased the frequency in the inflamed uterus, similarly to ACh, PGF_2α_, PGI_2_ and LTC_4_^30,32,33,39^. Moreover, the participation of CGRPR in the influence of CGRP on uterine inflammation, shown in the current study, may constitute the basis for the development of drugs (agonists, antagonists) to increase the contractility of uteri with inflammation. This may contribute to an improvement in the effectiveness of treatment and prevent postpartum diseases of the reproductive tract and, thus, to better fertility and economic results on farms.

## Conclusions

Severe acute endometritis did not change the total population of nerve fibers, including the CGRP-like immunoreactive fibers and increased the CLR protein expression in pig myometrium. In the inflamed uterus, CGRP by CGRPR increases the contractile amplitude in the myometrium and reduces this parameter in the endometrium/myometrium, and increases the frequency in the endometrium/myometrium, which suggests a possible regulatory function for CGRP in the contractility of the uterus during spontaneous inflammation. The current findings offer an important basis for the future study of CGRPR components expression and mechanisms of changes in their amounts and, as a result, the eventual significance of CGRPR in the prevention and treatment of disturbances in myometrium contractility in the setting inflammation of the uterus.

## Materials and methods

### Animals

Fifteen gilts (female pig after puberty before farrowing, Large White × Landrace, age 7–8 months, body weight/BW/90–120 kg) from the “Wronie” breeding farm (Wronie, Poland) were used in the experiment. There were no reproductive disturbances in these animals (vaginal discharges did not occur and the second estrous cycle was regular). Behavioral estrus was determined by a tester boar. Transport of gilts from a farm to the animal house (University of Warmia and Mazury, Olsztyn, Poland) took place three days before the start of the research. The animals were kept in individual pens (an area: approx. 5 m^2^) under 14.5 ± 1.5 h of natural daylight and 9.5 ± 1.5 h of night, and 18 ± 2 °C of temperature. They were fed commercial diets and had access to water ad libitum. The study procedures were conducted according to the relevant Polish and EU regulations in the field of Animal Protection and Welfare (Leg. Decree 26/2014 implementing EU directive 2010/63/EU), and were approved by the Local Ethics Committee (Consent no. 65/2015).

### Study procedures

The gilts were allocated (randomly), on day 3 of the second estrous cycle (day 0 of the research), into three groups: *Escherichia coli (E. coli)*, saline (SAL), control (CON) (five animals in a particular group).

The research procedures have been reported in detail^39^. The premedication was evoked using atropine (0.05 mg/kg BW; Atropinum sulf. WZF, Warszawskie Zakłady Farmaceutyczne Polfa S.A., Poland), azaperone (2 mg/kg BW; Stresnil, Janssen Pharmaceutica, Beerse, Belgium) and ketamine hydrochloride (10 mg/kg BW; Ketamina, Biowet, Puławy, Poland). Ketamine hydrochloride (supplementary doses: 1 mg/kg BW every 5 min) was also used for induction and maintenance of general anesthesia. After median laparotomy into each uterine horn in the *E. coli* group 50 ml of *E. coli* suspension (strain O25:K23/a/:H1; Department of Microbiology, National Veterinary Research Institute, Puławy, Poland), containing 10^9^ colony-forming units/ml were injected. In the SAL group, 50 ml of saline solution was injected. In the gilts from the CON group, only median laparotomy was made. The animals were left untreated in the time from surgery to euthanasia. The euthanasia was performed on day 8 of the experiment (the expected day 11 of the estrous cycle) using an overdose of sodium pentobarbital and the uteri were harvested. For real-time reverse transcriptase-polymerase chain reaction (real-time RT-PCR) and Western blot analyses, fragments of the horn were collected from three parts: paraoviductal, middle, and paracervical. Using a scalpel blade and a dissecting microscope, endometrial and myometrial layers were separated. The fragments of myometrium about the thickness of the entire layer were snap-frozen in liquid nitrogen and stored at − 80 °C for real-time RT-PCR and Western blot analysis. For the immunofluorescent method, the fragments of horn from three parts were divided into smaller pieces and placed in a 4% paraformaldehyde solution (pH 7.4) for 24 h. After fixation, the pieces were rinsed in 0.1 M phosphate-buffered saline (PBS, pH 7.4) and cryoprotected in an 18% buffered solution of sucrose (pH 7.4) until sectioning. To measure the uterine contractility, fragments of the horn from its middle part were placed on ice and transported to the laboratory (within 5 min following collection).

#### RNA extraction, and real-time RT-PCR

Total RNA was isolated from myometrial tissues. They were homogenized in a TRI Reagent solution (Invitrogen, Thermo Fisher Scientific, USA) using a FastPrep 24 homogenizer (MP Biomedicals, LCC, USA). For phase separation, a BCP reagent (Molecular Research Center Inc., USA) was used, and the RNA was then purified by using an RNeasy Mini Kit (QIAGEN, USA) in accordance with the manufacturer's instructions. RNA was stored until further use at − 80 °C in RNase-free water with the addition of RNAse Inhibitor (Applied Biosystems, Thermo Fisher Scientific, USA). The quality and quantity of extracted RNA were determined by the use of NanoDrop 1000 (Thermo Fisher Scientific, USA) and Agilent 2100 Bioanalyzer (Agilent Technologies, USA). RNA with an RNA Integrity number ranging from 7.0 to 9.6 was used in real-time RT-PCR.

Real-time RT-PCR was carried out by the use of TaqMan tests (Table 1) and a one-step PCR Master mix (Applied Biosystems). Each reaction (10 μl) contained: 15 ng of total RNA in a volume of 3 µl, 5 μl 2× TaqMan RT-PCR Mix, 0.25 μl 40× TaqMan RT Enzyme Mix, 0.5 μl 20× TaqMan Gene Expression Assays and 1.25 μl RNase-free water (Applied Biosystems). The real-time RT-PCR reaction was performed in duplicates in 384-well plates using the following conditions: reverse transcription for 15 min at 48 °C, initial denaturation for 10 min at 95 °C, followed by 45 cycles of 15 s of denaturation at 95 °C and then 1 min of annealing at 60 °C, in an ABI Prism 7900HT system (Applied Biosystems). The negative control was prepared by replacing the RNA template with RNase-free water. Data obtained were analyzed by the use of the Miner method^71^. The NormFinder algorithm was utilized to choose the most stable housekeeping gene among: β-actin (ACTB), hypoxanthine–guanine phosphoribosyl transferase (HPRT) and glyceraldehyde-3-phosphate dehydrogenase (GAPDH)^72^. The best stability value was determined for the combination of ACTB and GAPDH genes (0.171). The expression levels for each target gene were normalized relative to the geometric mean of ACTB and GAPDH gene expression.

**Table 1:** TaqMan assays used in the experiment.

Symbol	Name	Assay no.
*CALCRL*	*Calcitonin receptor-like*	*Ss03394019_g1*
*GAPDH*	*Glyceraldehyde-3-phosphate dehydrogenase*	*Ss03375435_u1*
*ACTB*	*β-Actin*	*Ss03376081_u1*
*HPRT*	*Hypoxanthine guanine phosphoribosyl transferase*	*Ss03388274_m1*

#### Western blot analysis

The myometrial tissues were homogenized on ice with a cold buffer (composition: 50 mmol/l Tris–HCl, pH 8.0; 150 mmol/l NaCl; 1% Triton X-100, 10 mg/ml aprotinin, 52 mmol/l leupeptin, 1 mmol/l pepstatin A, 1 mmol/l EDTA, 1 mol/l PMSF) and centrifuged (2500 g, at 4 °C, for 10 min). The supernatants were centrifuged (17,500×*g*, at 4 °C, for 1 h), and the collected supernatants were then stored at − 80 °C. The Bradford method was used to estimate the protein content^73^. Protein extracts (20 μg) were dissolved in a sodium dodecyl sulfate (SDS) gel-loading buffer (composition: 50 mmol/l Tris–HCl, pH 6.8; 4% SDS, 20% glycerol and 2% β-mercaptoethanol), heated (at 95 °C, for 4 min) and separated by 10% SDS–polyacrylamide gel electrophoresis. The separated proteins were then electro-blotted onto nitrocellulose membrane (0.22 μm) in transfer buffer (composition: 20 mmol/l Tris–HCl buffer, pH 8.2; 150 mmol/l glycine, 20% methanol, 0.05% SDS). To block the non-specific bindings, membranes were incubated with 5% fat-free dry milk in a TBS-T buffer (at 21 °C, for 1.5 h). Next, they were incubated (at 4 °C, for 18 h) with primary antibody rabbit CRLR/CGRPR1 polyclonal antibody (dilution: 1:500, cat. no. bs-1860R), from Bioss Antibodies Inc. After rinsing in TBS-T buffer, the membranes were incubated (at 21 °C, for 1 h) with biotinylated goat anti-rabbit IgG (dilution: 1:3000, cat. no. PK-6101, Vectastain Elite ABC-HRP Kit, Vector Labs, Burlingame, CA, USA). To visualize antibody binding, incubation (for 3–4 min) with a mixture of 3,3ʹ-diaminobenzidine tetrachloride (cat. no. D5637, Sigma Aldrich, St. Louis, MO, USA) and H_2_O_2_ in Tris-buffered saline (pH 7.2) was performed. To demonstrate the specificity of the primary antibody utilized, it was excluded from the analysis (the negative control). Mice and porcine duodenal proteins were used as the positive control. Images were gained and quantified by a Quantity-One system (VersaDoc 4000 M imaging system, Bio-Rad Laboratories, Hercules, CA, USA). The density of bands was normalized vs the protein content of GAPDH.

#### Immunofluorescence

The pieces of horns of uteri were cut using a cryostat (Reichert-Jung, Nußloch, Germany). Sections (thickness10 μm) were stained using the single-immunofluorescent method to estimate immunoreactivity to CLR, and the double-labeling immunofluorescence method to determine PGP9.5- and CGRP-like IR nerve fibers^74^. In short, uterine sections after drying (at 21 °C, for 30 min) and rinsing (0.1 M PBS, pH 7.4, three times, each for 15 min) were incubated (at 21 °C, for 1 h) with buffered blocking mixture (composition: 0.1 M PBS, 10% normal goat serum/MP Biomedicals, Solon, OH, USA/, 0.1% bovine serum albumin /Sigma-Aldrich, St. Louis, MO, USA/, 0.05% Thimerosal /Sigma-Aldrich, St. Louis, MO, USA/, 1% Triton X-100/Sigma-Aldrich, St. Louis, MO, USA/, 0.01% sodium azide). After washing (as given above), the sections were incubated (at 21 °C, for 18 h) in a humidity chamber, with a primary antibody against CRLR/CGRPR1 (dilution: 1:200), the same as for Western blotting. On the next day, the sections were washed (as given above) and incubated with biotinylated anti-rabbit IgG (dilution: 1:1000, cat. no. AP132B, Chemicon International, Temecula, CA, USA) (at 21 °C, for 1 h), and next with carbocyanine 3 (CY3)-conjugated streptavidin (dilution: 1:9000, cat. no. 016160084, Jackson ImmunoResearch Labs, West Grove, PA, USA) (at 21 °C, for 1 h). The sections were also incubated (at 21 °C, for 18 h) with antibodies against the PGP9.5 (dilution 1:800, polyclonal rabbit, cat. no. 104004, Abcam, UK) and CGRP (dilution: 1:1600, polyclonal guinea pig, cat. no. T-5027, BMA Biomedicals, Augst, Switzerland). Following rinsing (as given above), the sections were incubated with biotinylated anti-rabbit IgG (dilution: 1:1000, cat. no. AP132B, Chemicon International, Temecula, CA, USA) (at 21 °C, for 1 h), and then with CY3-conjugated streptavidin (the same as given above) and fluorescein isothiocyanate (FITC)-conjugated donkey anti-guinea pig IgG (dilution: 1:800, cat. no. 706095148, Jackson ImmunoResearch Labs, West Grove, PA, USA) (at 21 °C, for 1 h) to visualize the antibody combinations: PGP9.5/CGRP. Next, the washed sections were coverslipped in carbonate-buffered glycerol (pH 8.6). To perform the negative controls the primary antibodies were omitted. As a positive control, sections of the porcine duodenum were used. Immunoreactivity was assessed using the microscope with epi-fluorescence and appropriate filters (Olympus BX51, Olympus Consilio Sp. z o. o., Warsaw, Poland). Immunostained structures were analyzed and photographed using an Olympus BX51 microscope (Olympus, Consilio Sp. z o.o., Warsaw, Poland) equipment with epi-fluorescence and the appropriate filter sets for FITC (B1 module, excitation filter 450–480 nm, barrier filter 515 nm) and CY3 (G1 module excitation filter 510–550 nm, barrier filter 590 nm). The density of nerve fibers in the myometrium was evaluated according to the method given earlier^12^ using computer software (Image Processing and Analysis in Java, v1.53 m). In brief, the counting of these structures was performed in five randomly chosen microscopic observation fields (each: 0.1 mm^2^) in the myometrium of six sections of each uterus. To prevent double-counting the same fibers, the uterine sections to be assessed were at a distance of at least 100 µm.

#### Preparation of uterine strips and contractility measurement

The strips (approximate size 3 × 5 mm) of myometrium and endometrium/myometrium were used to study contractile function^31^. After rinsing in saline, they were mounted between two stainless steel hooks in an organ bath with a capacity of 10 ml (Radnoti Unit Tissue Organ Bath System type 159920, Germany) under 5 mN tension. The Krebs–Ringer solution (composition /mM/l/: NaCl, 120.3; KCl, 5.9; CaCl_2_, 2.5; MCl_2_, 1.2; NaHCO_3_, 15.5; glucose, 11.5; pH 7.4) placed in the bath was at 37 °C and was constantly saturated (mixture of 95% O_2_, 5% CO_2_).

**Figure 7:**
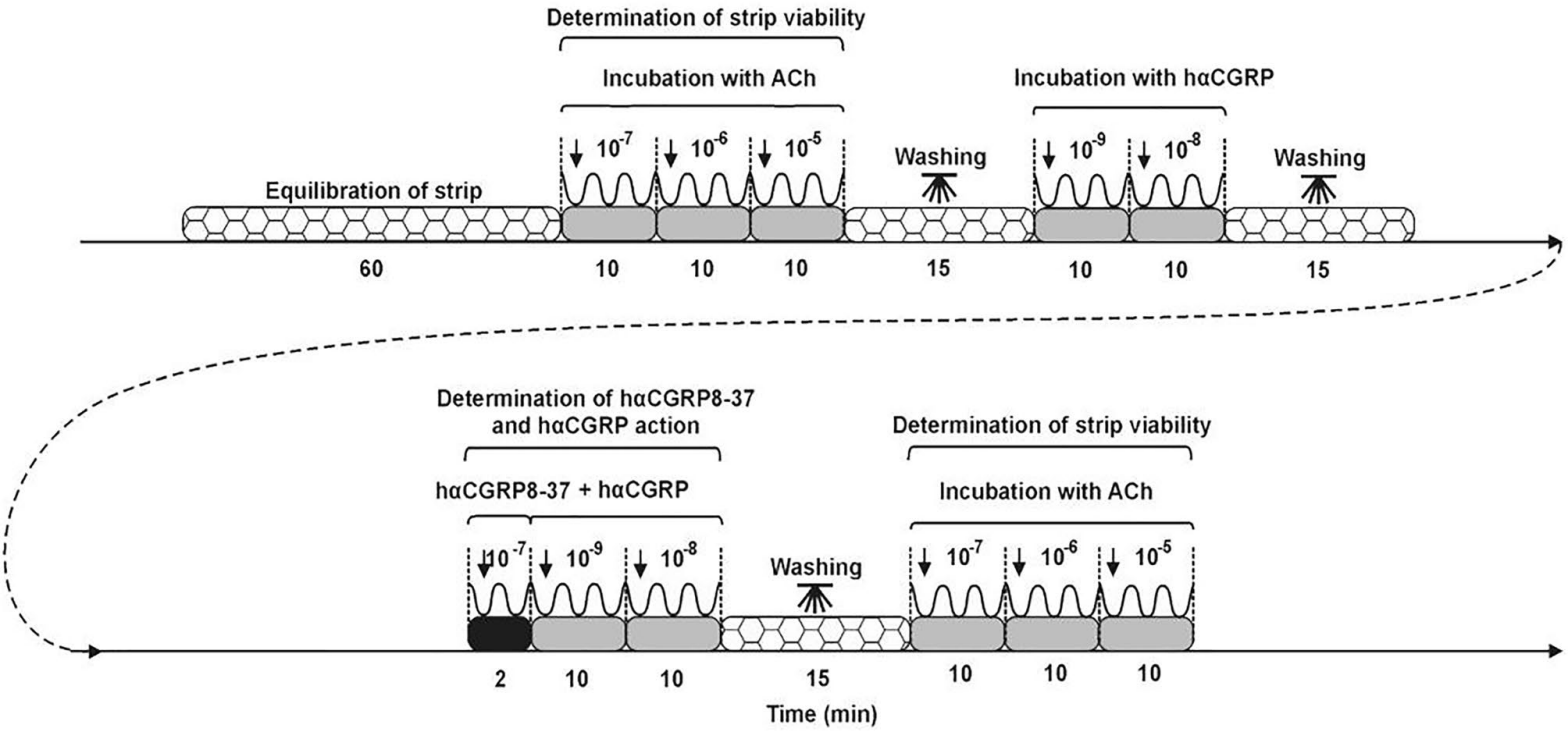
Schematic diagram of uterine strip treatment. *ACh* acetylcholine, *hαCGRP* human α-calcitonin gene-related peptide, *hαCGRP8–37* human α-calcitonin gene-related peptide receptor. Concentrations of these factors are given in moles.

Amplitude (the difference between the minimum and maximum values for a single contraction /mN/) and frequency (the number of peaks) of the strips were registered by a force–displacement transducer and analyzed in a computer with PowerChart software (Chart v5, scope v5, AD Instruments). The uterine strip treatments are depicted in Fig. 7. To estimate the viability of strips and their usefulness for further study, strips were influenced by ACh (doses: 10^–7^, 10^–6^, 10^–5^ M, cat. no. A6625, Sigma, St. Louis, MO, USA) which was reported earlier^39^. Following this, hαCGRP (doses: 10^–9^, 10^–8^ M, cat. no. ab142518, Abcam, UK) was used. The action of a particular dose of hαCGRP was measured for 10 min. Subsequently, the strips were under the influence of CGRPR antagonist—hαCGRP8–37 (dose: 10^–7^ M, cat. no. ab142492, Abcam, UK) for 2 min and hαCGRP (doses: 10^–9^, 10^–8^ M) was then added, and the effects of both substances were registered for 10 min. After each measurement, the strip was washed (in PBS, three times). After completing the measurements, the viability of strips was determined again using ACh (doses as given above). Only results registered from strips in which the discrepancies under the influence of ACh at the beginning and end of the study were less than 20% were included in the statistical analysis. The ACh doses were used previously^30,31^. To estimate the hαCGRP and hαCGRP8–37 doses, the initial research was performed in which healthy pig uteri were treated with CGRP (doses: 10^–10^, 10^–9^, 10^–8^ M) alone and together with an antagonist (doses: 10^–9^, 10^–8^, 10^–7^ M). As a result, it was found that hαCGRP at doses of 10^–9^ and 10^–8^ M more effectively influenced the contractile parameters and that hαCGRP8–37 at a dose of 10^–7^ M statistically significantly changed hαCGRP-affected the contractile parameters (data not present).

### Statistical analyses

Mean (± SEM) total numbers of PGP9.5- and CGRP-like IR nerve fibers, the numbers of these fibers around particular myometrial structures, as well as mRNA and protein CLR levels were counted for particular groups. To determine differences in the frequency of CGRP-like IR nerve fibers as part of the total population of PGP9.5-like IR nerve fibers, the total number of PGP9.5-like IR fibers in each group was set to 100% and the number of CGRP-like IR fibers was expressed as a percentage (mean ± SEM) of the total population of PGP9.5-like IR fibers. The mean (± SEM) values of amplitude and frequency counted for a particular group before the addition of substances (pre-treatment period) were accepted as 100%. The influences of substances were expressed as the percentage (mean ± SEM) values of these parameters measured before their use. The analysis of contractile function concerned the comparisons between mean values before and following each treatment in each group, as well as the mean values between groups in response to the same treatment. The statistical significances between the obtained data were evaluated by the Bonferroni test (ANOVA, InStat Graph Pad, San Diego, CA). Three thresholds (*P < 0.05, **P < 0.01, ***P < 0.001) were used to indicate statistically significant differences.

### Ethical approval

The studies presented in the manuscript were carried out in accordance with the ARRIVE guidelines. All study procedures were approved by the Local Ethics Committee for Experiments on Animals (University of Warmia and Mazury in Olsztyn, Poland; Consent no. 65/2015). The guidelines in EU Directive 2010/63/EU for animal experiments were included.

## Supplementary Information

Supplementary Figures.

## Data Availability

The datasets used and/or analysed during the current study available from the corresponding author on reasonable request.

## References

[1] Katafuchi T, Yasue H, Osaki T, Minamin N (2009). Calcitonin receptor-stimulating peptide: Its evolutionary and functional relationship with calcitonin/calcitonin gene-related peptide based on gene structure. Peptides.

[2] Yallampalli C, Chauhan M, Endsley J, Sathishkumar K (2014). Calcitonin gene related family peptides: Importance in normal placental and fetal development. Adv. Exp. Med. Biol..

[3] Hay DL, Walker CS (2017). CGRP and its receptors. Headache.

[4] Russell FA, King R, Smillie SJ, Kodji X, Brain SD (2014). Calcitonin gene-related peptide: Physiology and pathophysiology. Physiol. Rev..

[5] Schou WS, Ashina S, Amin FM, Goadsby PJ, Ashina MJ (2017). Calcitonin gene-related peptide and pain: A systematic review. J. Headache Pain.

[6] Wasowicz K, Podlasz P, Czaja K, Łakomy M (2002). Uterus-innervating neurones of paracervical ganglion in the pig: Immunohistochemical characteristics. Folia Morphol. (Warsz).

[7] Bulc M, Całka J, Meller K, Jana B (2019). Endometritis affects chemical coding of the dorsal root ganglia neurons supplying uterus in the sexually mature gilts. Res. Vet. Sci..

[8] Tokushige N, Markham R, Russell P, Fraser IS (2006). Nerve fibres in peritoneal endometriosis. Hum. Reprod..

[9] Herweijer G, Kyloh M, Beckett EA, Dodds KN, Spencer NJ (2014). Characterization of primary afferent spinal innervation of mouse uterus. Front. Neurosci..

[10] Shew RL, Papka RE, McNeill DL (1992). Galanin and calcitonin gene-related peptide immunoreactivity in nerves of the rat uterus: Localization, colocalization, and effects on uterine contractility. Peptides.

[11] Gnanamanickam GJ, Llewellyn-Smith IJ (2011). Innervation of the rat uterus at estrus: A study in full-thickness, immunoperoxidase-stained whole-mount preparations. J. Comp. Neurol..

[12] Rytel L, Gonkowski S, Janowski T, Wojtkiewicz J, Pomianowski A (2019). The neurochemical characterization of parasympathetic nerve fibres in the porcine wall under physiological conditions and after exposure to bisphenol A (BPA). Neurotoxicity Res..

[13] Chan KK, Robinson G, Broughton Pipkin F (1997). Differential sensitivity of human nonpregnant and pregnant myometrium to calcitonin gene-related peptide. J. Soc. Gynecol. Investig..

[14] Naghashpour M, Rosenblatt MI, Dickerson IM, Dahl GP (1997). Inhibitory effect of calcitonin gene-related peptide on myometrial contractility is diminished at parturition. Endocrinology.

[15] Shew RL, Papka RE, McNeill DL, Yee JA (1993). NADPH-diaphorase-positive nerves and the role of nitric oxide in CGRP relaxation of uterine contraction. Peptides.

[16] Anouar A, Schirar A, Germain G (1998). Relaxant effect of the calcitonin gene-related peptide (CGRP) on the nonpregnant and pregnant rat uterus. Comparison with vascular tissue. Naunyn Schmiedebergs Arch. Pharmacol..

[17] Khomasuridze P, Bekaia TG, Bekaia GL (2009). Main factors determining the functional state of pregnant rat's uterus. Georgian Med. News.

[18] Dong YL (1999). Involvement of calcitonin gene-related peptide in the modulation of human myometrial contractility during pregnancy. J. Clin. Investig..

[19] Yallampalli C, Gangula PR, Kondapaka S, Fang L, Wimalawansa S (1999). Regulation of calcitonin gene-related peptide receptors in the rat uterus during pregnancy and labor and by progesterone. Biol. Reprod..

[20] Thota C, Yallampalli C (2005). Progesterone upregulates calcitonin gene-related peptide and adrenomedullin receptor components and cyclic adenosine 3'5'-monophosphate generation in Eker rat uterine smooth muscle cell line. Biol. Reprod..

[21] Naghashpour M, Dahl G (2000). Sensitivity of myometrium to CGRP varies during mouse estrous cycle and in response to progesterone. Am. J. Physiol. Cell Physiol..

[22] Pokabla MJ, Dickerson IM, Papka RE (2002). Calcitonin gene-related peptide-receptor component protein expression in the uterine cervix, lumbosacral spinal cord, and dorsal root ganglia. Peptides.

[23] Tummaruk P, Kesdangsakonwut S, Prapasarakul N, Kaeoket K (2010). Endometritis in gilts: Reproductive data, bacterial culture, histopathology, and infiltration of immune cells in the endometrium. Comp. Clin. Pathol..

[24] Sheldon IM, Cronin J, Goetze L, Donofrio G, Schuberth H-J (2009). Defining postpartum uterine disease and the mechanisms of infection and immunity in the female reproductive tract in cattle. Biol. Reprod..

[25] Mordak R, Stewart PA (2015). Periparturient stress and immune suppression as a potential cause of retained placenta in highly productive dairy cows: Examples of prevention. Acta Vet. Scand..

[26] Pascottini OB, LeBlanc SJ (2020). Modulation of immune function in the bovine uterus peripartum. Theriogenology.

[27] Wiebe M (2021). In vitro effects of lipopolysaccharides on bovine uterine contractility. Reprod. Domest. Anim..

[28] Heppelmann M (2016). The effect of puerperal uterine disease on histopathologic findings and mRNA expression of proinflammatory cytokines of the endometrium in dairy cows. Theriogenology.

[29] Sheldon IM, Owens SE, Turner ML (2017). Innate immunity and the sensing of infection, damage and danger in the female genital tract. J. Reprod. Immunol..

[30] Kucharski J (2007). The influence of inflammatory process on prostaglandin F2α contractile activity in porcine uterus. J. Anim. Feed Sci..

[31] Jana B (2010). Participation of prostaglandin E2 in contractile activity of inflamed porcine uterus. Acta Vet. Brno.

[32] Jana B, Jaroszewski J, Czarzasta J, Włodarczyk M, Markiewicz W (2013). Synthesis of prostacyclin and its effect on the contractile activity of the inflamed porcine uterus. Theriogenology.

[33] Jana B, Jaroszewski JJ, Czarzasta J, Markiewicz W (2015). The influence of leukotrienes C4 and D4 on the contractility of an inflamed porcine uterus. Theriogenology.

[34] Heppelmann M (2018). Effects of oxytocin and PGF2α on uterine contractility in cows with and without metritis—An in-vitro study. Anim. Reprod. Sci..

[35] Jana B, Całka J (2020). Endometritis changes the neurochemical characteristics of the caudal mesenteric ganglion neurons supplying the gilt uterus. Animals (Basel).

[36] Miciński B, Jana B, Całka J (2021). Endometritis decreases the population of uterine neurons in the paracervical ganglion and changes the expression of sympathetic neurotransmitters in sexually mature gilts. BMC Vet. Res..

[37] Meller KA, Całka J, Kaczmarek M, Jana B (2018). Expression of alpha and beta adrenergic receptors in the pig uterus during inflammation. Theriogenology.

[38] Jana B, Całka J (2021). Role of beta-adrenergic receptor subtypes in pig uterus contractility with inflammation. Sci. Rep..

[39] Jana B, Całka J, Bulc M, Piotrowska-Tomala KK (2020). Participation of acetylcholine and its receptors in the contractility of inflamed porcine uterus. Theriogenology.

[40] Jana B, Całka J, Palus K, Sikora M (2020). *Escherichia coli*-induced inflammation changes the expression of acetylcholine receptors (M2R, M3R, and α-7 nAChR) in pig uterus. J. Vet. Res..

[41] Jana B, Całka J, Palus K (2020). Inflammation changes the expression of neuropeptide Y receptors in the pig myometrium and their role in the uterine contractility. PLoS ONE.

[42] Jana B, Całka J, Czajkowska M (2020). The role of somatostatin and its receptors (sstr2, sstr5) in the contractility of gilt inflamed uterus. Res. Vet. Sci..

[43] Palus K, Całka J, Jana B (2021). Alterations in the relative abundance of the vasoactive intestinal peptide receptors (VPAC1 and VPAC2) and functions in uterine contractility during inflammation. Anim. Reprod. Sci..

[44] Jana B, Całka J, Miciński B (2021). Regulatory influence of galanin and GALR1/GALR2 receptors on inflamed uterus contractility in pigs. Int. J. Mol. Sci..

[45] Verma N, Rettenmeier AW, Schmitz-Spanke S (2011). Recent advances in the use of *Sus scrofa* (pig) as a model system for proteomic studies. Proteomics.

[46] De Winter PJJ, Verdonck M, de Kruif A, Devriese LA, Haesebrouck F (1992). Endometritis and vaginal discharge in the sow. Anim. Reprod. Sci..

[47] Sharma H (2018). Innervation changes induced by inflammation in the murine vagina. Neuroscience.

[48] Makowska K, Gonkowski S (2018). The influence of inflammation and nerve damage on the neurochemical characterization of calcitonin gene-related peptide-like immunoreactive (CGRP-LI) neurons in the enteric nervous system of the porcine descending colon. Int. J. Mol. Sci..

[49] Hobara N (2004). Distribution of adrenomedullin-containing perivascular nerves in the rat mesenteric artery. Peptides.

[50] Edvinsson L, Goadsby PJ, Uddman R (2001). Amylin: Localization, effects on cerebral arteries and on local cerebral blood flow in the cat. Sci. World J..

[51] Katafuchi T (2003). Calcitonin receptor-stimulating peptide, a new member of the calcitonin gene-related peptide family. Its isolation from porcine brain, structure, tissue distribution, and biological activity. J. Biol. Chem..

[52] Dong YL (2004). Involvement of calcitonin gene-related peptide in control of human fetoplacental vascular tone. Am. J. Physiol. Heart Circ. Physiol..

[53] Hagner S (2001). Immunohistochemical detection of calcitonin gene-related peptide receptor (CGRPR)-1 in the endothelium of human coronary artery and bronchial blood vessels. Neuropeptides.

[54] Choksi T (2002). Comparison of the expression of calcitonin receptor-like receptor (CRLR) and receptor activity modifying proteins (RAMPs) with CGRP and adrenomedullin binding in cell lines. Br. J. Pharmacol..

[55] Kroeger I (2009). The neuropeptide calcitonin gene-related peptide (CGRP) prevents inflammatory liver injury in mice. J. Hepatol..

[56] Thota C, Gangula PPR, Dong YL, Yallampalli C (2003). Changes in the expression of calcitonin receptor-like receptor, receptor activity-modifying protein (RAMP) 1, RAMP2, and RAMP3 in rat uterus during pregnancy, labor, and by steroid hormone treatments. Biol. Reprod..

[57] Minatani A (2016). Activation of calcitonin gene-related peptide signaling through the prostaglandin E2-EP1/EP2/EP4 receptor pathway in synovium of knee osteoarthritis patients. J. Orthop. Surg. Res..

[58] Nikitenko LL (2001). Differential and cell-specific expression of calcitonin receptor-like receptor and receptor activity modifying proteins in the human uterus. Mol. Hum. Reprod..

[59] Dong YL, Vegiraju S, Chauhan M, Yallampalli C (2003). Expression of calcitonin gene-related peptide receptor components, calcitonin receptor-like receptor and receptor activity modifying protein 1, in the rat placenta during pregnancy and their cellular localization. Mol. Hum. Reprod..

[60] Tsujikawa K (2007). Hypertension and dysregulated proinflammatory cytokine production in receptor activity-modifying protein 1-deficient mice. Proc. Natl. Acad. Sci. U.S.A..

[61] Chauhan M (2022). Calcitonin gene related peptide, adrenomedullin and adrenomedullin2 function in uterine artery during human pregnancy. Endocrinology.

[62] Chauhan M, Gangula PR, Wimalawansa SJ, Yallampalli C (2004). Studies on the effects of the N-terminal domain antibodies of calcitonin receptor-like receptor and receptor activity-modifying protein 1 on calcitonin gene-related peptide-induced vasorelaxation in rat uterine artery. Biol. Reprod..

[63] Holzer P (1988). Local effector functions of capsaicin-sensitive sensory nerve endings: Involvement of tachykinins, calcitonin gene-related peptide and other neuropeptides. Neuroscience.

[64] Clifton MS (2007). Role of calcitonin receptor-like receptor in colonic motility and inflammation. Am. J. Physiol. Gastrointest. Liver Physiol..

[65] Casey ML, Smith J, Alsabrook G (1997). Activation of adenylyl cyclase in human myometrial smooth muscle cells by neuropeptides. J. Clin. Endocrinol. Metab..

[66] Upton PD (1997). Expression of adrenomedullin (ADM) and its binding sites in the rat uterus: Increased number of binding sites and ADM messenger ribonucleic acid in 20-day pregnant rats compared with nonpregnant rats. Endocrinology.

[67] Shew RL, Papka RE, McNeill DL (1990). Calcitonin gene-related peptide in the rat uterus: Presence in nerves and effects on uterine contraction. Peptides.

[68] Ma W, Eisenach JC (2003). Intraplantar injection of a cyclooxygenase inhibitor ketorolac reduces immunoreactivities of substance P, calcitonin gene-related peptide, and dynorphin in the dorsal horn of rats with nerve injury or inflammation. Neuroscience.

[69] Jana B, Andronowska A, Kucharski J (2000). Nitric oxide mediates an inflammatory effect of *Escherichia coli* in the porcine uterus. Pol. J. Vet. Sci..

[70] Jana B, Kucharski J, Dzienis A, Deptuła K (2007). Changes in prostaglandin production and ovarian function in gilts during endometritis induced by *Escherichia coli* infection. Anim. Reprod. Sci..

[71] Zhao S, Fernald RD (2005). Comprehensive algorithm for quantitative real-time polymerase chain reaction. J. Comput. Biol..

[72] Andersen CL, Jensen JL, Ørntoft TF (2004). Normalization of real-time quantitative reverse transcription-PCR data: A model-based variance estimation approach to identify genes suited for normalization, applied to bladder and colon cancer data sets. Cancer Res..

[73] Bradford MM (1976). A rapid and sensitive method for the quantitation of microgram quantities of protein utilizing the principle of protein-dye binding. Anal. Biochem..

[74] Majewski M, Heym C (1991). The origin of ovarian neuropeptide Y (NPY)-immunoreactive nerve fibres from the inferior mesenteric ganglion in the pig. Cell Tissue Res..

