# Supplementary material for: Involvement of the calcitonin gene-related peptide system in the modulation of inflamed uterus contractile function in pigs
Source: Sci Rep. 2022 Nov 9;12:19146. doi: 10.1038/s41598-022-23867-6 (PMC9646719; doi:10.1038/s41598-022-23867-6)
Supplement: Supplementary file 1 — Supplementary Figures. [file 41598_2022_23867_MOESM1_ESM.docx]

**Supplementary material**


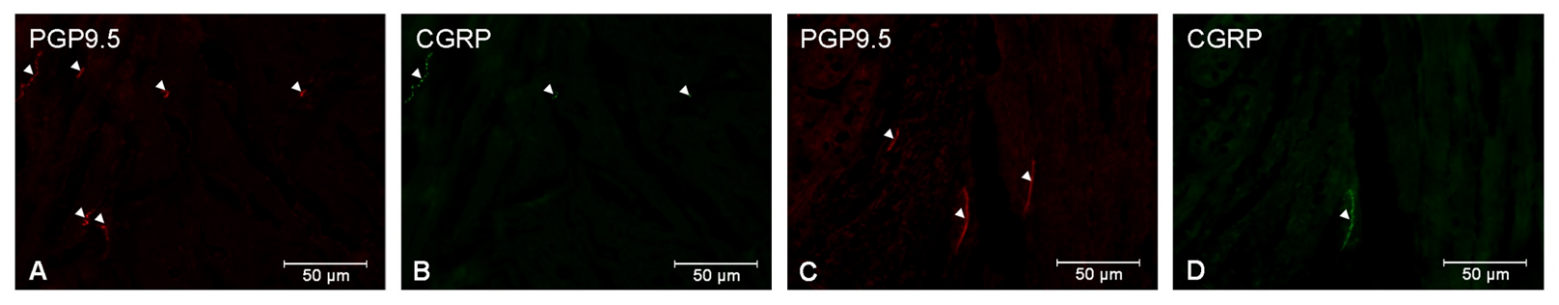


**Supplementary Figure 1.** The protein gene product (PGP)9.5- and calcitonin gene-related peptide (CGRP)-like immunoreactive (IR) nerve fibers in the porcine myometrium and relevant positive control. Pictures show the PGP9.5- and CGRP-like IR nerve fibers in porcine myometrium (A, B, respectively) and duodenum (C, D, respectively). Arrowhead – nerve fiber


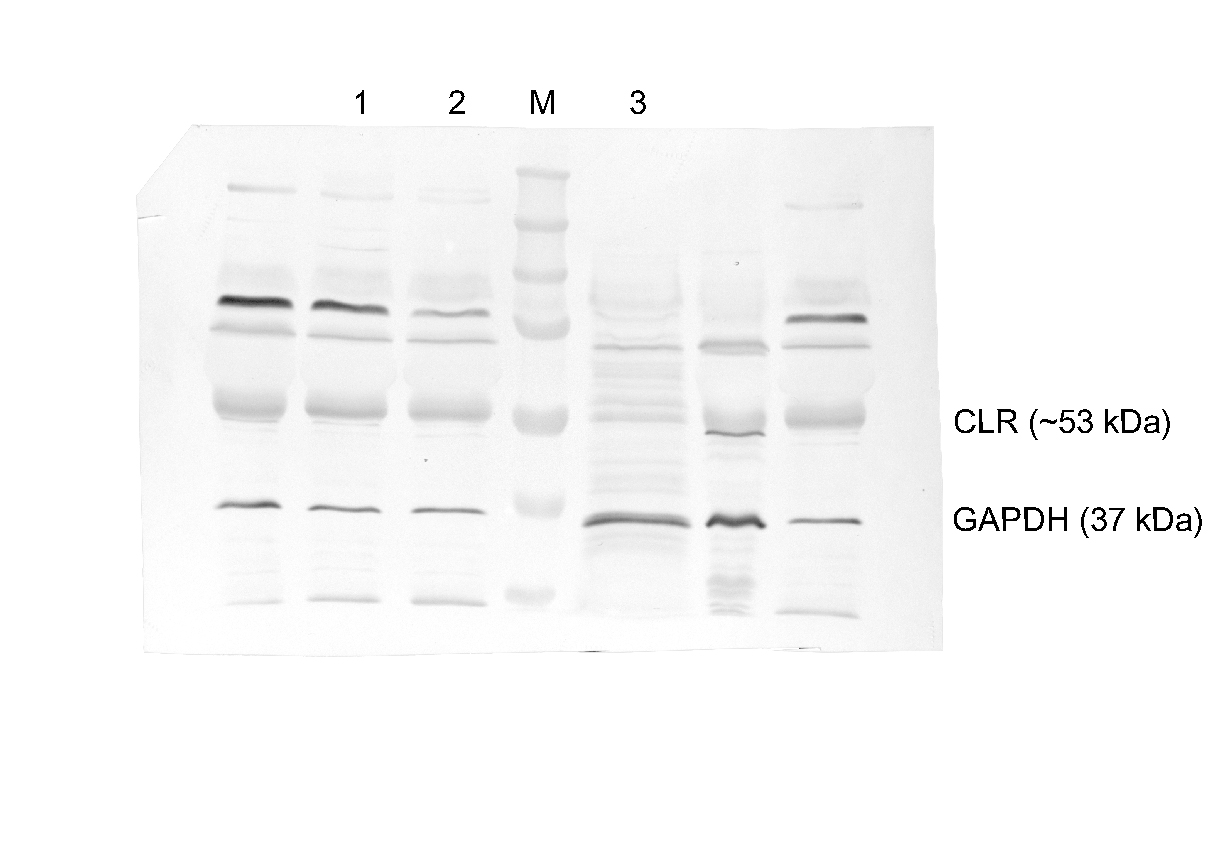


**Supplementary** **Figure 2.** Western blot analysis of calcitonin receptor-like receptor (CLR) in porcine myometrium and relevant positive control. For CLR antibody bands at approximately 53 kDa are visible in porcine myometrium (line 1) and porcine (line 2) and mouse (line 3) duodenum. The density of bands was normalized in relation to glyceraldehyde-3-phosphate dehydrogenase (GAPDH). M - marker.


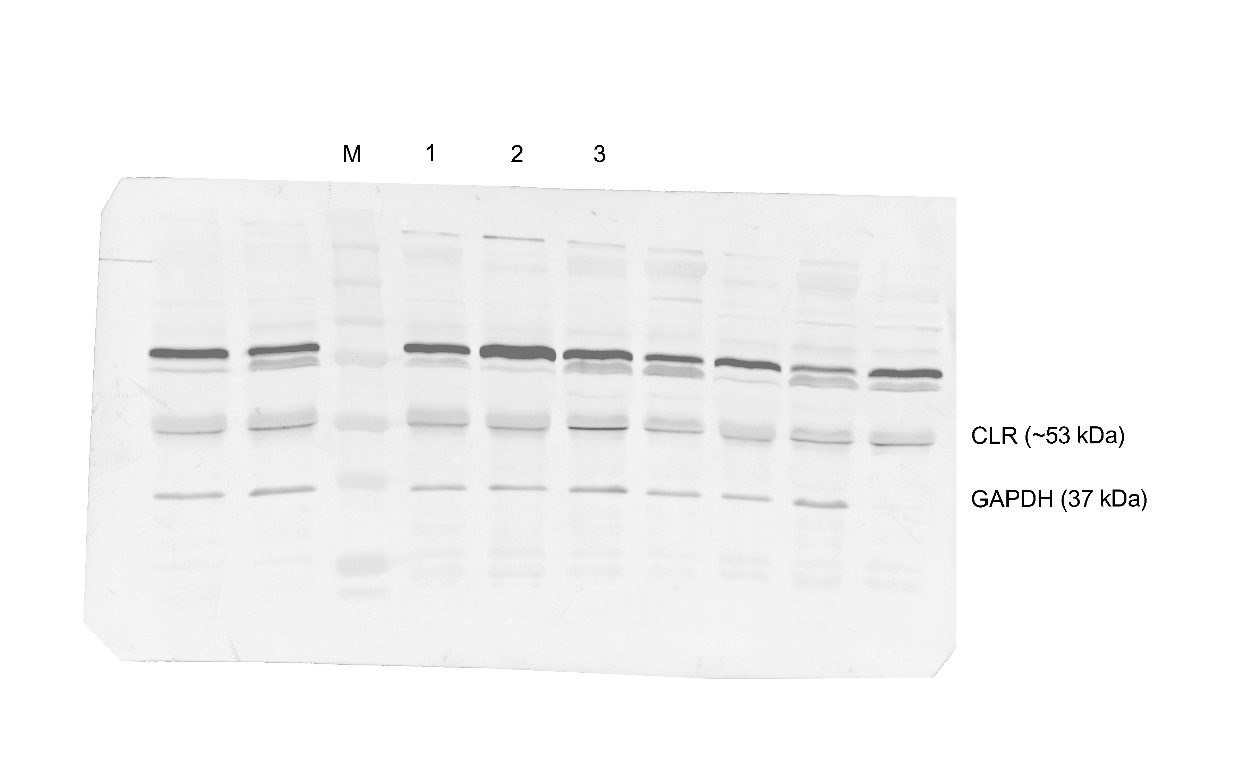


**Supplementary Figure 3.** Bot shows representative bands of calcitonin receptor-like receptor (CLR) protein expression in the myometrial layer of gilts from the control (CON; line 1), saline (SAL; line 2) and *E. coli* (*E. coli*; line 3) groups, estimated by Western blot analysis. The density of bands was normalized in relation to glyceraldehyde-3-phosphate dehydrogenase (GAPDH). M - marker.


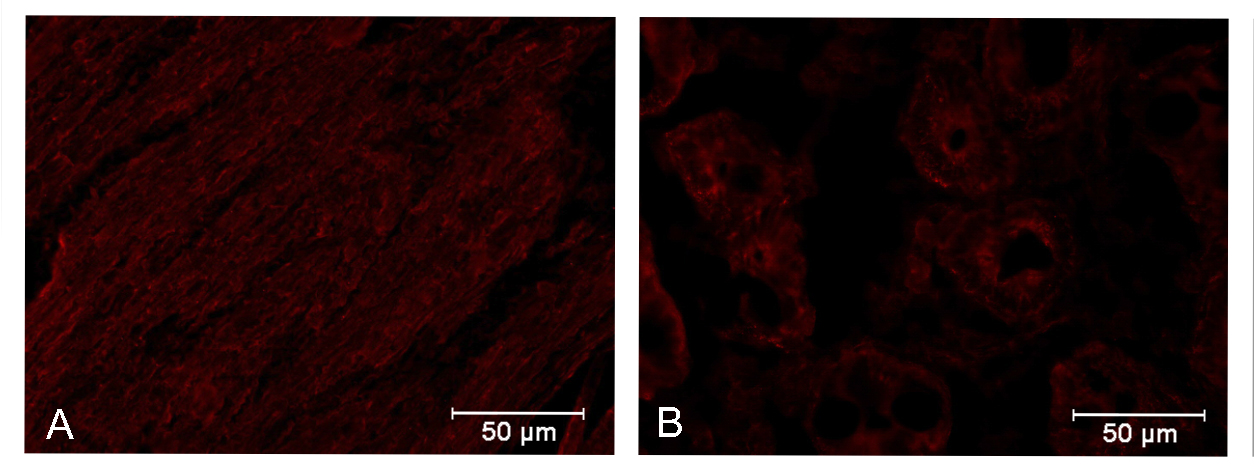


**Supplementary Figure 4.** The calcitonin receptor-like receptor (CLR)-like immunoreactivity in porcine myometrium and relevant positive control. Pictures show the CLR in porcine myometrium (A) and duodenum (B).
